# Relationship between Menopausal Hormone Therapy and Oral Cancer: A Cohort Study Based on the Health Insurance Database in South Korea

**DOI:** 10.3390/jcm11195848

**Published:** 2022-10-02

**Authors:** Jin-Sung Yuk, Bo Young Kim

**Affiliations:** 1 Department of Obstetrics and Gynecology, Sanggye Paik Hospital, School of Medicine, Inje University, Seoul 01757, Korea; 2 Department of Otolaryngology, Sanggye Paik Hospital, School of Medicine, Inje University, Seoul 01757, Korea

**Keywords:** diagnosis, estrogen, head and neck cancer, menopausal hormone therapy, menopause, oral cancer, squamous cell carcinoma, tibolone

## Abstract

The association between the development of oral cavity cancer and sex hormones is unclear and inconsistent. This study aimed to evaluate the relationship between menopausal hormone therapy (MHT) and oral cavity cancer in menopausal women in Korea. In this retrospective cohort study, data were provided by the Korean National Health Insurance Service regarding a screening examination conducted from 1 January 2002 to 31 December 2019. Postmenopausal patients aged ≥40 years were considered, including 333,072 women in the MHT group and 847,558 women in the non-MHT group. Participants were divided into MHT types (tibolone, combined estrogen plus progestin by manufacturer, estrogen, combined estrogen plus progestin by physician, and topical estrogen), and the risk factors for oral cavity cancer development were analyzed. There was no significant association between smoking, alcohol consumption, age at menarche, and age at menopause with oral cavity cancer in postmenopausal women. However, the oral estrogen (hazard ratio [HR]: 1.633; 95% confidence interval [CI]: 1.35–1.976) and tibolone groups (HR: 1.633; 95% CI: 1.35–1.976) were associated with an elevated risk of oral cavity cancer. The results of this study suggest that MHT increases the risk of oral cavity cancer in postmenopausal women.

## 1. Introduction

Oral cavity cancer is the 6th most common cancer [1] and the 18th most commonly diagnosed malignancy worldwide, accounting for 2.0% of all cancer cases in 2020, according to the GLOBACAN database [2]. The incidence is higher in men than in women [3,4]. As the risk factors for oral cavity cancer, a history of smoking and alcohol consumption is important [1,5]. Several studies, however, have found that non-smoking and non-drinking (NSND) patients present with head and neck cancer (HNC), mostly oral cavity cancer and mainly in older women [6,7,8,9,10]. NSND elderly female patients have poor disease-specific survival outcomes [8,9]. Few studies have attempted to understand the development of oral cavity cancer among female patients who do not have these traditional risk factors by focusing on hormonal factors [11,12,13,14]. Furthermore, it has been noted that the difference in oral cancer incidence between men and women decreases with age [3,4,6,7,8,9,10], with a few studies suggesting that sex hormones play a role in oral cavity cancer development because of the high incidence rate among older women [11,12,13,14].

Estrogen and progesterone hormone receptor expression in oral cavity cancer has been reported in recent studies. Estrogen receptor (ER) α was not expressed in normal oral mucosa, but was in oral cavity cancer [15]. Coexpression of ER α, β, and progesterone receptor (PgR) was frequent in HNC [16]. In HNC, ER and epidermal growth factor receptor (EGFR) are associated and are involved in cancer development and disease progression [17]. In oral cavity cancer, EGRF has been reported to be involved in the mitogen-activated protein (MAPK) and focal adhesion kinase (FAK) pathways via the ER [17,18,19] Although ER and PgR expression has been demonstrated in oral cavity cancer, the role of sex hormones in tumor development remains uncertain.

There are few studies on the association between sex hormones and the development of oral cavity cancer. Suba suggested that estrogen deficiency was a risk factor for oral cavity cancer in postmenopausal women [11]. McCarthy et al. reported that menopausal hormone therapy (MHT) lowered the incidence of HNC or esophageal squamous cell carcinoma, and that earlier age at menopause was a risk factor [12]. Another study found that exogenous estrogen stimulated HNC proliferation in vitro via the ER [17]. However, no association was observed in a study by Corrao et al. [20]. The relationship between MHT and cancer risk remains unclear and inconsistent.

The aim of this cohort study was to evaluate the relationship between menopausal hormone therapy and oral cavity cancer using data from the Health Insurance Databases in South Korea.

## 2. Materials and Methods

### 2.1. Database

Under the national healthcare system of South Korea, the provision of national health insurance is legally mandated for most of the Korean population (approximately 51 million people) [21]. Therefore, the National Health Insurance Service (NHIS) of South Korea maintains patient records for most of the diseases apart from a few exceptional cases such as cosmetic surgery (diagnosis code, operative procedure code, name of prescription drugs, type of medical insurance, income quintile, region, and hospital types) (“Health Security System,” n.d.). A regular nationwide health screening is implemented by the NHIS for the health management of adults among the Korean population. Therefore, the NHIS manages the information of the Korean population who underwent the screening, including information regarding body mass index (BMI), parity, age at menarche, age at menopause, smoking, alcohol consumption, and physical exercise (“Health Security System,” n.d.) This retrospective cohort study was based on the data provided by the NHIS on the national health screening conducted and insurance from 1 January 2002 to 31 December 2019.

### 2.2. Selection of Participants

In the selection of patients and outcomes, the International Classification of Diseases, 10th revision (ICD-10) and the Korea Health Insurance Medical Care Expenses (2012, 2016, 2019 version) were used as the reference criteria. For the extraction of the case and control group, only women aged 40 years and above who were recorded as postmenopausal in the medical history taken between 2002 and 2011 were extracted as study participants. The study participants were followed up through 2019. The MHT group included women who had been prescribed MHT for >6 months. The start date of MHT was defined as the first day of MHT prescription. If two or more hormones were taken alternately during the cohort period, the definition was based on the last hormone taken for ≥6 months. The control group was defined as women who had never used MHT between 2002 and 2019. The start date for the control group was defined as the first date recorded as postmenopausal in the medical history. If this date was missing from the record, it was defined as June 30 of the year of the national health screening.

The following participants were excluded from both the case and control groups:

(i) A participant confirmed as menopausal in 2002, to consider wash out;
(ii) If a participant had one or more cancer-related diagnosis code (any C code) within 180 days after inclusion;
(iii) If a participant had one or more disease of the oral cavity (K0, K1) within 180 days after inclusion.

### 2.3. Outcome

A case of confirmatory diagnosis of oral cancer was defined as a patient with three or more visits to a hospital with an oral cancer diagnosis code (C00–C14) for primary diagnosis or secondary diagnosis.

### 2.4. Variables

The MHT types were classified as follows: tibolone, combined estrogen plus progestin by manufacturer (CEPM), estrogen, combined estrogen plus progestin by physician (CEPP), and topical estrogen (Supplementary Table S1). Independent variables included age, BMI, history of smoking and alcohol consumption, Charlson Comorbidity Index (CCI), socioeconomic status (SES), physical exercise, region, parity, age at menarche, age at menopause, and period from menopause to inclusion, based on the date of inclusion. Age was grouped into ten-year intervals, and the definition of BMI was based on the Asia-Pacific perspective [22]. When the type of medical insurance was medical aid, the participant was categorized as low SES, and the location of the hospital was categorized as either a metropolitan or rural region. The CCI was calculated using the diagnosis codes at hospital visits from one year before the date of study participation to the study participation date [23]. Since there was no option of “0” in the questions of medical history taking, the parity variable was classified into 0 or no response, 1, 2, and 3 or more. The smoking variable was classified into never, past, and current, whereas alcohol consumption was classified according to the number of drinks per week. Physical exercise was classified according to the weekly frequency of exercise for ≥30 min.

### 2.5. Statistical Analysis

For all statistical analyses in this study, SAS Enterprise Guide 6.1 (SAS Institute Inc., Cary, NC, USA) was used.

Statistical significance was defined as a *p*-value of ≤0.05. A two-sided test was performed for all statistics in this study. Continuous variables are expressed in terms of median values (25 percentile, 75 percentile), and categorical variables are expressed as numbers (percentage). The Cox proportional hazard model was used for the calculation of adjustment for various confounding factors (age group, BMI, SES, region, CCI, parity, age at menarche, age at menopause, smoking and alcohol consumption, physical exercise, and period from menopause to inclusion). For the sensitivity test for verification of the robustness of the study results, only cases prescribed by a gynecologist were selected from the MHT group for calculation of the Cox proportional hazard model. In this study, the listwise deletion method was used for handling missing values.

### 2.6. Ethics

This study was approved by the Institutional Review Board of Inje University Sangye Paik Hospital (approval No.: SGPAIK-2020-08-002). Data used in this study were provided after de-identification of participants from the NHIS data. Therefore, since the study participants were unidentifiable, there was no harm to the individuals included in the data. Additionally, this study did not require informed consent from the patients included in the data according to the Bioethics and Safety Act of South Korea. Data analysis in this study could only be conducted on the NHIS server according to the NHIS privacy policy, and raw data other than the result values could not be exported. Moreover, according to the NHIS policy, information on the MHT manufacturer (in the absence of generics) was provided in combination with other hormones.

## 3. Result

**Figure 1:**
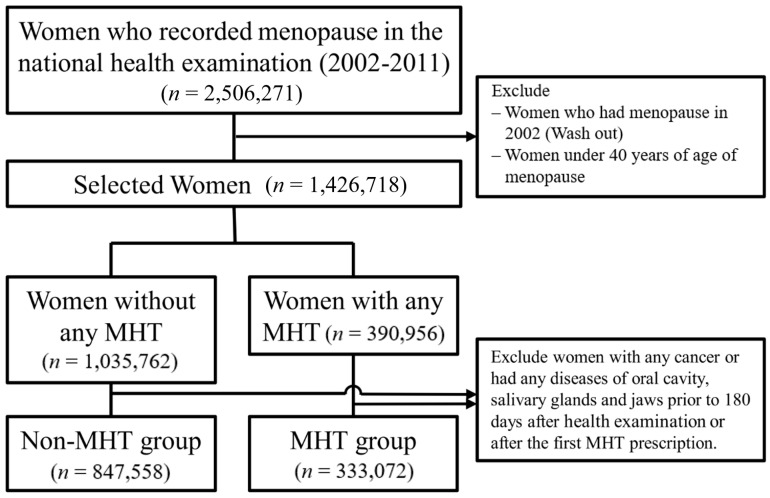
Flowchart to select case-control according to MHT using the Korea National Health Insurance Data, 2002–2019. MHT, menopausal hormone therapy.

From 2002 to 2011, overall 2,506,271 women confirmed as postmenopausal during the national health screening of South Korea were initially selected. Among them, only postmenopausal patients aged ≥40 years between 2003 and 2011 were considered. Finally, 333,072 women in the MHT group and 847,558 women in the non-MHT group were included as study participants (Figure 1).

When classified according to the hormone used, there were 167,674, 111,494, 46,278, 5776, and 1850 patients in the tibolone, CEPM, oral estrogen, CEPP, and topical estrogen groups, respectively (Table 1). The mean age of all women participated in this study was 56 (52–62) years and the mean BMI value was 23.8 (21.9–25.9) kg/m^2^. Table 1 presents the detailed information on the characteristics of the women who participated in this study.

In the MHT group, the period of hormone prescription within 5 years accounted for the highest proportion (76.8%), followed by 5–9 years (17.1%), and ≥10 years (6.2%). Table 2 outlines the characteristics specific to the MHT group.

The number of participants diagnosed with oral cancer in each group were as follows: 1782 (0.2%), 308 (0.2%), 159 (0.1%), 121 (0.3%), 15 (0.3%), and 4 (0.2%) in the non-MHT, tibolone, CEPM, oral estrogen, CEPP, and topical estrogen groups, respectively.

**Figure 2:**
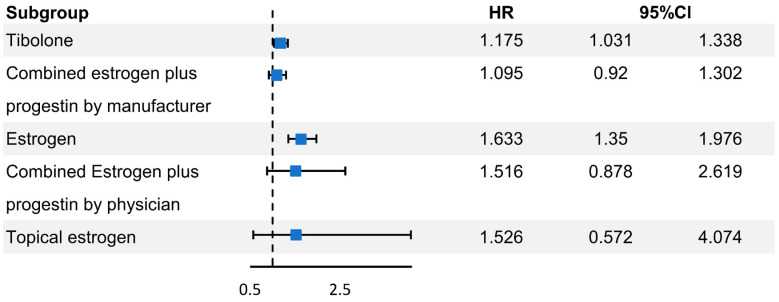
Hazard ratio for risk of oral cancer according to MHT.

In the Cox proportional hazard analysis adjusted for variables such as age, BMI, SES, region area, CCI, parity, age at menarche, age at menopause, smoking, alcohol, physical exercise, and period from menopause to inclusion, the number of participants diagnosed with oral cancer increased in the tibolone (hazard ratio [HR]: 1.175, 95% confidence interval [CI]: 1.031–1.338) and oral estrogen groups (HR: 1.633, 95% CI: 1.35–1.976). However, there was no significant change in the CEPM (HR: 1.095, 95% CI: 0.92–1.302), CEPP (HR: 1.516, 95% CI: 0.878–2.619), and topical estrogen groups (HR: 1.526, 95% CI: 0.572–4.074) (Figure 2).

In the Cox proportional hazard analysis, old age (≥70 years) (HR: 3.219, 95% CI: 2.408–4.303), low SES (HR: 1.465, 95% CI: 1.173–1.828), rural area (HR: 1.707, 95% CI: 1.535–1.897), and high parity (≥3) (HR: 2.979, 95% CI: 2.349–3.777) increased the incidence of oral cancer, whereas physical exercise (3–4 times per week) lowered the risk of oral cancer (HR: 0.725, 95% CI: 0.612–0.859). However, BMI, age at menarche, age at menopause, smoking, and alcohol consumption showed no significant correlation with oral cancer incidence (Supplementary Table S2).

Considering the age variable, oral estrogen increased the risk of oral cancer in all age groups over 50 years. The highest risk was observed especially in those over 70 years of age (HR: 2.931, 95% CI: 1.715–5.011).

## 4. Discussion

The incidence of oral cancer is higher in men than in women; however, the gap narrows with increasing age [3,4,6,7,8,9,10]. Although smoking and alcohol consumption are important risk factors for oral cavity cancer, these risk factors are not observed in some patients. NSND patients with oral cavity cancer are mainly observed among older women. Farshadpour et al. compared 195 NSND patients with HNC and 4209 patients with HNC retrospectively. Of the NSND patients with HNC, 142 (73%) were women, with a mean age of 73 years (median 76, range 20–97) [6]. Similarly, in a retrospective study on 287 patients with oral cavity cancer, 70 (24.4%) patients had NSND, of whom, 53 (18.5%) were women (M:F; 1:3.12). Moreover, of the 39 (13.6%) NSND patients, 28 (9.75%) were women over the age of 70 [8]. In our study, an increased risk was observed with increasing age (≥70 years) (HR: 3.219; 95% CI: 2.408–4.303), whereas smoking and alcohol consumption were not associated with the risk of oral cavity cancer in postmenopausal women.

As age increases, the difference in the incidence rate between men and women decreases, and in the absence of risk factors, the incidence rate increases among older women. This supports the idea that sex hormones contribute to the development of oral cavity cancer. From this point of view, in the study by Suba, the mean age at menopause was lower in patients with oral cavity cancer than in those in the control groups. Furthermore, the period from menopause to oral cavity cancer diagnosis was significantly shorter in the group with early menopause than in the group with late menopause (13.4 years vs. 23.5 years). These findings suggest that estrogen deficiency is a risk factor for oral cavity cancer in postmenopausal women [11]. McCarthy et al. reported that earlier age at menopause was a risk factor for esophageal cancer and that MHT lowered the incidence of HNC [12]. In a cohort study with 297 cases of HNC, MHT significantly lowered the risk of HNC (HR: 0.77; 95% CI: 0.62–0.96) [14]. In a case control study with 253 participants, an inverse association was observed between MHT and HNC (OR: 0.7; 95% CI: 0.4–1.2) [24]. Langevin et al. discovered a borderline protective effect of an inverse dose response on HNC with time [13].

Interestingly, in this study, MHT increased the risk for oral cavity cancer in postmenopausal women. We found that oral cavity cancer was increased in the oral estrogen (HR: 1.633; 95% CI: 1.35–1.976) and tibolone groups (HR: 1.633; 95% CI: 1.35–1.976). Our findings suggest that sex hormones play a role in the etiology of oral cavity cancer as well as other hormone-related cancers, such as ovarian, endometrial, breast, and prostate cancer, with oral estrogen and tibolone therapy having a significant association.

Despite these, the mechanism of sex hormones in the etiology of oral cavity cancer remains unknown. Several studies have found ER and PgR in oral cavity cancer. Through a molecular and immunocytochemical approach, Lukits et al. discovered frequent expression of ER and PgR in HNC. ER expression was more common in oral cavity cancer than in laryngeal and hypopharyngeal cancer (58.2% vs. 46.5%) [16]. Egloff et al. reported that in an HNC cell line (including 23 oral cavity cancers), ERα was 95% positive, ERβ was 44% positive, and an estrogen activated MAPK signal pathway. Furthermore, exogenous estrogen promoted HNC proliferation though the ER in cancer cell growth and in vitro invasion [17]. Their findings are consistent with the results of this study.

According to Chang et al., functional ER is expressed in oral squamous cell carcinoma and plays an important role in cell growth promotion. This indicates that ER could be a therapeutic target in oral squamous cell carcinoma [18]. Furthermore, previous studies also reported that tamoxifen targeting ERα in HNC inhibited cell proliferation and invasion [16,19,25,26]. In the study by Ishida et al., ER antagonist agents caused the inhibition of invasion and cell adhesion and affected the MAPK pathway [19]. Tamoxifen, an ER antagonist, reduces ER α phosphorylation in oral cavity cancer cells, which is linked to FAK activation [18,19]. As a result, MAPK is also decreased, which inhibits cancer cell invasion [19]. Kim et al. discovered that the combination of tamoxifen and cisplatin was more effective in oral squamous cell carcinoma cell lines in terms of cytotoxicity and apoptosis [25].

There was no association between HNC and parity in a case-control study by Langevin et al. involving 149 women with HNC [13]. Another study with a woman with laryngeal cancer showed that lower parity had a protective effect [27]. Our study presents that high parity (≥3) is a risk factor for oral cavity cancer (HR: 2.979, 95% CI: 2.349–3.777). Progesterone and estrogen are secreted at higher levels during pregnancy than in non-pregnancy. Therefore, compared to primipara women, women with higher parity are exposed to more estrogen and progesterone. Our results show that high parity increased the risk of oral cancer, suggesting that sex hormones are a risk factor for oral cancer. Although there is a difference between endogenous sex hormones and artificial sex hormones (MHT), there is a consistency in that sex hormones are risk factors for oral cancer. Moreover, similar to other study findings, age at menarche and age at menopause did not show significance in our results [13,14,28].

This study has several strengths. First, this study is one of the largest studies in terms of population on the relationship between MHT and oral cavity cancer. Second, our data included all participants from all regions of Korea because the data were provided by the NHIS. Third, we analyzed the HR for variable values (age, BMI, socioeconomic status, region, CCI, parity, age at menarche, age at menopause, smoking, alcohol consumption, physical exercise, and period from menopause).

There are also some limitations of this study. First, we used data based on the diagnosis codes without reviewing medical records. The data of misdiagnosed patients may have been inadvertently included. Second, MHT is a broad spectrum of conjugated estrogens with a wide variety of doses depending on the patient’s condition such as hysterectomy. Some patients take more than one type of MHT. If two or more MHTs were used in this study, they were included in the MHT group in which patients had been taking MHT for ≥6 months. We did not investigate the impact of multiple MHT intake. Third, in this study, we did not analyze the impact of therapy duration on the incidence of oral cancer. Fourth, this study did not include data on human papillomavirus, which is a common risk factor for cervical cancer and oral cavity cancer. Estrogens can cause endometrial cancer when used alone, whereas progesterone is used to prevent endometrial cancer [29]; however, as hysterectomy patients have no need for endometrial protection, oral estrogen alone is administered [29]. Therefore, patients who have undergone hysterectomy for cervical cancer (including cervical intraepithelial neoplasia) are more likely to develop oral cancer caused by human papillomavirus and are more likely to be treated with oral estrogen alone. For this reason, it is assumed that the risk of oral cavity cancer is higher in the oral estrogen group. This result is similar to that in the tibolone group. Because tibolone does not cause breast cancer, it can be used in women with or without a uterus [30]. Therefore, the tibolone group may also have an increased risk of oral cavity cancer, although not as much as the oral estrogen group.

## 5. Conclusions

In conclusion, we observed an association between MHT, especially oral estrogen and tibolone, and oral cavity cancer development in postmenopausal women. Although further research is warranted, this finding presents useful information in the investigation of the etiology of oral cavity cancer with MHT in postmenopausal women.

## Figures and Tables

**Table 1:** Characteristics of women according to menopausal hormone exposure status at recruitment, Korea National Health Insurance Data, 2002–2019.

	Non-MHT	Tibolone	Combined Estrogen Plus Progestin by Manufacturer	Oral Estrogen	Combined Estrogen Plus Progestin by Physician	Topical Estrogen	Total
Number of women	847,558	167,674	111,494	46,278	5776	1850	1,180,630
Median age (years)	57 (52–64)	53 (50–57)	52 (50–56)	52 (49–57)	54 (51–59)	53 (50–57)	56 (52–62)
Age at inclusion (years)							
40–49	76,449 (9)	29,682 (17.7)	25,628 (23)	12,251 (26.5)	1017 (17.6)	397 (21.5)	145,424 (12.3)
50–59	422,920 (49.9)	110,026 (65.6)	74,109 (66.5)	26,006 (56.2)	3449 (59.7)	1131 (61.1)	637,641 (54)
60–69	249,788 (33.3)	25,030 (15.2)	10,908 (9.9)	6661 (14.8)	1154 (20.5)	294 (16.1)	293,835 (27.3)
≥70	98,401 (11.6)	2936 (1.8)	849 (0.8)	1360 (2.9)	156 (2.7)	28 (1.5)	103,730 (8.8)
Median BMI (kg/m^2^)	24 (22.1–26.1)	23.5 (21.8–25.4)	23.1 (21.5–25)	23.7 (22–25.7)	23.3 (21.6–25.2)	23.7 (22.1–25.7)	23.8 (21.9–25.9)
BMI (kg/m^2^)							
<18.5	15,309 (1.8)	2831 (1.7)	2172 (2)	653 (1.4)	111 (1.9)	36 (2)	21,112 (1.8)
18.5–22.9	284,993 (34.3)	67,199 (40.5)	50,000 (45.2)	16,989 (37)	2395 (41.8)	678 (36.9)	422,254 (36.3)
23–24.9	220,558 (26.5)	46,239 (27.8)	29,811 (26.9)	12,817 (27.9)	1609 (28.1)	493 (26.9)	311,527 (26.8)
25–29.9	274,470 (33)	45,588 (27.5)	26,512 (23.9)	13,864 (30.2)	1484 (25.9)	575 (31.3)	362,493 (31.2)
≥30	36,225 (4.4)	4180 (2.5)	2215 (2)	1560 (3.4)	126 (2.2)	53 (2.9)	44,359 (3.8)
SES							
Mid-high SES	813,432 (96)	161,857 (96.5)	108,792 (97.6)	45,008 (97.3)	5632 (97.5)	1800 (97.3)	1,136,521 (96.3)
Low SES	34,126 (4)	5817 (3.5)	2702 (2.4)	1270 (2.7)	144 (2.5)	50 (2.7)	44,109 (3.7)
Region							
Urban area	258,929 (30.6)	53,369 (31.8)	38,421 (34.5)	14,944 (32.3)	2992 (51.8)	856 (46.3)	369,511 (31.3)
Rural area	588,629 (69.4)	114,305 (68.2)	73,073 (65.5)	31,334 (67.7)	2784 (48.2)	994 (53.7)	811,119 (68.7)
CCI							
0	559,530 (66)	114,818 (68.5)	79,524 (71.3)	32,404 (70)	4017 (69.5)	1219 (65.9)	791,512 (67)
1	162,498 (19.2)	32,158 (19.2)	19,768 (17.7)	8312 (18)	1063 (18.4)	340 (18.4)	224,139 (19)
≥2	125,530 (14.8)	20,698 (12.3)	12,202 (10.9)	5562 (12)	696 (12)	291 (15.7)	164,979 (14)
Parity (years)							
0 or no response	147,119 (17.4)	27,049 (16.1)	14,604 (13.1)	9955 (21.5)	1185 (20.5)	415 (22.4)	200,327 (17)
1	50,951 (6)	14,569 (8.7)	11,578 (10.4)	3582 (7.7)	433 (7.5)	150 (8.1)	81,263 (6.9)
2	543,362 (73.3)	110,609 (72.7)	76,976 (74.6)	28,103 (67.5)	3565 (68.8)	1102 (66.1)	763,717 (73.1)
≥3	106,126 (12.5)	15,447 (9.2)	8336 (7.5)	4638 (10)	593 (10.3)	183 (9.9)	135,323 (11.5)
Age at menarche (years)							
<13	141,163 (16.7)	24,863 (14.9)	16,246 (14.7)	8611 (18.9)	1095 (19.1)	356 (19.5)	192,334 (16.4)
≥13	702,034 (83.3)	141,448 (85.1)	94,584 (85.3)	37,051 (81.1)	4631 (80.9)	1471 (80.5)	981,219 (83.6)
Age at menopause (years)							
40–44	105,727 (12.5)	20,387 (12.2)	12,780 (11.5)	10,088 (21.8)	754 (13.1)	372 (20.1)	150,108 (12.7)
45–49	244,173 (28.8)	54,782 (32.7)	37,370 (33.5)	16,454 (35.6)	1867 (32.3)	668 (36.1)	355,314 (30.1)
50–54	424,057 (54.8)	79,815 (51.5)	53,930 (51.8)	17,500 (39.7)	2704 (50.8)	699 (40.2)	578,705 (53.4)
≥55	73,601 (8.7)	12,690 (7.6)	7414 (6.6)	2236 (4.8)	451 (7.8)	111 (6)	96,503 (8.2)
Smoking							
Never	773,580 (96.3)	151,051 (93.6)	100,720 (93.3)	42,217 (94.9)	5314 (95.5)	1705 (96.4)	1,074,587 (95.6)
Past	8338 (1)	2818 (1.7)	1996 (1.8)	613 (1.4)	74 (1.3)	24 (1.4)	13,863 (1.2)
Current	21,499 (2.7)	7482 (4.6)	5206 (4.8)	1664 (3.7)	174 (3.1)	39 (2.2)	36,064 (3.2)
Alcohol (per week)							
None	684,433 (84.8)	125,500 (77.2)	82,043 (75.6)	35,634 (79.4)	4627 (82.5)	1470 (81.9)	933,707 (82.6)
~2/week	104,804 (13)	31,424 (19.3)	22,663 (20.9)	8054 (17.9)	859 (15.3)	289 (16.1)	168,093 (14.9)
3–6/week	12,893 (1.6)	4240 (2.6)	3088 (2.9)	851 (1.9)	86 (1.5)	28 (1.6)	21,186 (1.9)
Daily	4641 (0.6)	1340 (0.8)	794 (0.7)	340 (0.8)	36 (0.6)	7 (0.4)	7158 (0.6)
Physical exercise (per week)							
None	522,432 (64.7)	95,838 (59)	64,610 (59.5)	26,535 (59.2)	3153 (56.3)	940 (52.7)	713,508 (63.1)
1–2	135,837 (16.8)	31,225 (19.2)	21,254 (19.6)	8717 (19.4)	1111 (19.8)	387 (21.7)	198,531 (17.6)
3–4	76,024 (9.4)	18,907 (11.6)	12,844 (11.8)	5016 (11.2)	744 (13.3)	262 (14.7)	113,797 (10.1)
5–6	25,227 (3.1)	6174 (3.8)	4215 (3.9)	1604 (3.6)	223 (4)	72 (4)	37,515 (3.3)
Daily	48,487 (6)	10,261 (6.3)	5654 (5.2)	2962 (6.6)	373 (6.7)	123 (6.9)	67,860 (6)
Period from menopause to inclusion (years)							
<5	340,592 (40.2)	97,498 (58.1)	75,723 (67.9)	24,366 (52.7)	2977 (51.5)	937 (50.6)	542,093 (45.9)
5–9	182,521 (21.5)	38,929 (23.2)	22,305 (20)	11,632 (25.1)	1413 (24.5)	499 (27)	257,299 (21.8)
≥10	324,445 (38.3)	31,247 (18.6)	13,466 (12.1)	10,280 (22.2)	1386 (24)	414 (22.4)	381,238 (32.3)

BMI, Body mass index; CCI, Charlson comorbidity index; MHT, menopausal hormone therapy; SES, socioeconomic status.

**Table 2:** Characteristics of women prescribed menopausal hormone therapy, Korea National Health Insurance Data, 2002–2019.

MHT Characteristics	Tibolone	Combined Estrogen Plus Progestin by Manufacturer	Oral Estrogen	Combined Estrogen Plus Progestin by Physician	Topical Estrogen	Total MHT
Median duration (months)	25 (11–59)	25 (11–58)	15 (9–40)	16 (9–36)	13 (8–25)	23 (11–56)
Duration (years)						
<5	126,162 (75.2)	84,509 (75.8)	38,224 (82.6)	5026 (87)	1764 (95.4)	255,685 (76.8)
5–9.9	30,171 (18)	20,519 (18.4)	5513 (11.9)	575 (10)	82 (4.4)	56,860 (17.1)
≥10	11,341 (6.8)	6466 (5.8)	2541 (5.5)	175 (3)	4 (0.2)	20,527 (6.2)
Duration of previous other MHT (years)			0			
<5	163,170 (97.3)	109,728 (98.4)	45,619 (98.6)	4829 (83.6)	1830 (98.9)	325,176 (97.6)
5–9.9	4015 (2.4)	1621 (1.5)	586 (1.3)	691 (12)	19 (1)	6932 (2.1)
≥10	489 (0.3)	145 (0.1)	73 (0.2)	256 (4.4)	1 (0.1)	964 (0.3)
Last dosage of Tibolone (per day)						
1.25 mg	1602 (1)					
2.5 mg	165,898 (99)					
over 5 mg	156 (0.1)					
Prescribed specialty						
Gynecology	56,418 (33.6)	50,897 (45.6)	18,863 (40.8)	1343 (23.3)	452 (24.4)	127,973 (38.4)
Non-gynecology	111,256 (66.4)	60,597 (54.4)	27,415 (59.2)	4433 (76.7)	1398 (75.6)	205,099 (61.6)

MHT, menopausal hormone therapy.

## Data Availability

This article contains all of the data analyzed during this study. The data presented in this study are available on request from the corresponding author.

## Supplementary Materials

The following supporting information can be downloaded at: https://www.mdpi.com/article/10.3390/jcm11195848/s1, Table S1: Menopausal hormones by group; Table S2: Hazard ratios for oral cancer risk according to major variables, Korea National Health Insurance Data, 2002–2019.
