# Supplementary material for: Relationship between Menopausal Hormone Therapy and Oral Cancer: A Cohort Study Based on the Health Insurance Database in South Korea
Source: J Clin Med. 2022 Oct 2;11(19):5848. doi: 10.3390/jcm11195848 (PMC9572981; doi:10.3390/jcm11195848)
Supplement: Supplementary file 1 [file jcm-11-05848-s001.zip › jcm-1915512-supplementary.pdf]

Supplementary table S1. Menopausal hormones by group

MHT (generic name)	Brand name
Tibolone	
Tibolone 2.5 mg	Livial, Rabilone, Libolone, Libron, Live on, Liviem, Tiborisi, Tibolan, Tibiol, Pharmbio Korea Tibolone, Hyundai Tibolone
Combined estrogen plus progestin by manufacturer	
Estradiol Hemihydrate 1.03 mg, Drospirenone 2 mg	Angeliq
Estradiol Valerate 2 mg, Medroxyprogesterone Acetate 10 mg	Divina
Estradiol Valerate 1 mg, Medroxyprogesterone Acetate 2.5 mg	Indivina Tab 1 mg/2.5 mg
Estradiol Valerate 1 mg, Medroxyprogesterone Acetate 5 mg	Indivina Tab 1 mg/5 mg
Estradiol Valerate 2 mg, Medroxyprogesterone Acetate 2.5 mg	Indivina Tab 2 mg/2.5 mg
Estradiol Hemihydrate 2 mg, Norethisterone Acetate 1 mg	Cliane
Estradiol Hemihydrate 2.06 mg, Dydrogesterone 10 mg	Femoston 2/10
Estradiol Hemihydrate 1.03 mg, Dydrogesterone 10 mg	Femoston 1/10
Estradiol Hemihydrate 1.03 mg, Dydrogesterone 5 mg	Femoston Conti
Cyproterone Acetate 1 mg, Estradiol Valerate 2 mg	Climen
Estradiol Hemihydrate 1.03 mg, Norethisterone Acetate 0.5 mg	Esdiol-half
Estradiol Valerate 1.31 mg, Norethisterone Acetate 0.5 mg	Cliovelle
Estrogen	
Conjugated Estrogens 0.3 mg	Premina 0.3 mg
Conjugated Estrogens 0.625 mg	Premina 0.625 mg
Estradiol Valerate 1 mg	Progynova 1 mg
Estradiol Valerate 2 mg	Progynova 2 mg
Estradiol Hemihydrate 1 mg	Preda 1 mg
Combined estrogen plus progestin by physician [a]	
Progesterone Micronized 100 mg	Utrogestan 100 mg
Medroxyprogesterone Acetate 5 mg	Provera 5 mg
Medroxyprogesterone Acetate 10 mg	Provera 10 mg
Dydrogesterone 10 mg	Duphaston
Topical estrogen	
Estradiol Hemihydrate	Estreva gel, Climara patch, Divigel gel

MHT, menopausal hormone therapy

[a] This group used the progestin below and the estrogen group above simultaneously.

Supplementary table S2. Hazard ratios for oral cancer risk according to major variables, Korea National Health Insurance Data, 2002-2019

Variables	Formula 1[a]		Formula 2[b]	
	HR (95% CI) [a]	P-value	HR (95% CI) [b]	P-value
MHT				
Tibolone	1.189 (1.048-1.349)	0.007	1.175 (1.031-1.338)	0.015
Combined estrogen plus progestin by manufacturer	1.076 (0.908-1.276)	0.398	1.095 (0.92-1.302)	0.307
Oral estrogen	1.614 (1.337-1.947)	<0.001	1.633 (1.35-1.976)	<0.001
Combined estrogen plus progestin by physician	1.563 (0.923-2.647)	0.097	1.516 (0.878-2.619)	0.136
Topical estrogen	1.426 (0.534-3.807)	0.478	1.526 (0.572-4.074)	0.399
Age at inclusion (years)				
50~59	1.587 (1.325-1.902)	<0.001	1.464 (1.204-1.782)	<0.001
60~69	3.113 (2.581-3.754)	<0.001	2.316 (1.784-3.007)	<0.001
70~	4.514 (3.676-5.545)	<0.001	3.219 (2.408-4.303)	<0.001
BMI (kg/m2)				
<18.5	1.112 (0.82-1.507)	0.496	1.106 (0.813-1.506)	0.521
23-24.9	1.011 (0.907-1.127)	0.841	0.996 (0.892-1.113)	0.945
25-29.9	1.114 (1.008-1.232)	0.035	1.076 (0.97-1.192)	0.165
≥30	1.031 (0.832-1.276)	0.782	1.03 (0.83-1.279)	0.788
SES				
Low SES	1.491 (1.204-1.846)	<0.001	1.465 (1.173-1.828)	<0.001
Region				
Rural area	1.684 (1.52-1.865)	<0.001	1.707 (1.535-1.897)	<0.001
CCI				
1	1.181 (1.067-1.306)	0.001	1.165 (1.051-1.293)	0.004
≥2	1.132 (1.007-1.271)	0.037	1.118 (0.992-1.26)	0.067
Parity (years)				
0	2.348 (1.841-2.995)	<0.001	2.296 (1.792-2.942)	<0.001
2	1.049 (0.837-1.316)	0.677	1.024 (0.814-1.289)	0.839
≥3	3.076 (2.435-3.885)	<0.001	2.979 (2.349-3.777)	<0.001
Age at menarche (years)				
≥13	0.885 (0.778-1.007)	0.064	1.115 (0.977-1.273)	0.107
Age at menopause (years)				
45-49	1.134 (0.984-1.307)	0.083	1.158 (0.998-1.343)	0.053
50-54	1.071 (0.929-1.234)	0.347	1.171 (0.999-1.373)	0.051
55-	0.972 (0.797-1.187)	0.784	1.142 (0.908-1.438)	0.257
Smoking				

Supplementary table 2. *cont*

Past	0.882 (0.573-1.358)	0.569
Current	1.068 (0.834-1.368)	0.601
Alcohol (g/week)		
~2/week	0.999 (0.875-1.14)	0.983
3~6/week	0.88 (0.596-1.3)	0.521
Daily	0.656 (0.34-1.264)	0.208
Physical exercise (per week)		
1~2	0.932 (0.827-1.05)	0.249
3~4	0.725 (0.612-0.859)	<0.001
5~6	0.785 (0.595-1.036)	0.088
Daily	1.217 (1.052-1.409)	0.008
Period from menopause to inclusion (years)		
5~9	1.065 (0.929-1.222)	0.367
10~	1.324 (1.106-1.586)	0.002

BMI, Body mass index; CCI, Charlson comorbidity index; CI, confidence interval; HR, hazard ratio; MHT, menopausal hormone therapy; SES, socioeconomic status.

[a] HRs were adjusted for age group, body mass index, socioeconomic status, region, Charlson comorbidity index, parity, age at menarche, age at menopause.

[b] HRs were adjusted for age group, body mass index, socioeconomic status, region, Charlson comorbidity index, parity, age at menarche, age at menopause, smoking, alcohol, physical exercise, period from menopause to inclusion.
